# Learning gene regulatory networks from only positive and unlabeled data

**DOI:** 10.1186/1471-2105-11-228

**Published:** 2010-05-05

**Authors:** Luigi Cerulo, Charles Elkan, Michele Ceccarelli

**Affiliations:** 1 Department of Biological and Environmental Studies, University of Sannio, Benevento, Italy; 2 Biogem s.c.ar.l., Institute of Genetic Research "Gaetano Salvatore", Ariano Irpino (AV), Italy; 3 Department of Computer Science and Engineering, University of California, San Diego, CA, USA

## Abstract

**Background:**

Recently, supervised learning methods have been exploited to reconstruct gene regulatory networks from gene expression data. The reconstruction of a network is modeled as a binary classification problem for each pair of genes. A statistical classifier is trained to recognize the relationships between the activation profiles of gene pairs. This approach has been proven to outperform previous unsupervised methods. However, the supervised approach raises open questions. In particular, although known regulatory connections can safely be assumed to be positive training examples, obtaining negative examples is not straightforward, because definite knowledge is typically not available that a given pair of genes do not interact.

**Results:**

A recent advance in research on data mining is a method capable of learning a classifier from only positive and unlabeled examples, that does not need labeled negative examples. Applied to the reconstruction of gene regulatory networks, we show that this method significantly outperforms the current state of the art of machine learning methods. We assess the new method using both simulated and experimental data, and obtain major performance improvement.

**Conclusions:**

Compared to unsupervised methods for gene network inference, supervised methods are potentially more accurate, but for training they need a complete set of known regulatory connections. A supervised method that can be trained using only positive and unlabeled data, as presented in this paper, is especially beneficial for the task of inferring gene regulatory networks, because only an incomplete set of known regulatory connections is available in public databases such as RegulonDB, TRRD, KEGG, Transfac, and IPA.

## Background

Inferring the topology of gene regulatory networks is fundamental to understand the complexity of interdependencies among gene up and down regulation. Characterizing experimentally the transcriptional cis-regulation at a genome scale is still an expensive challenge, even for well-studied model organisms. In silico methods represent a promising direction that, through a reverse engineering approach, aim to extract gene regulatory networks from prior biological knowledge and available genomic and post-genomic data. Different model architectures to reverse engineer gene regulatory networks from gene expression data have been proposed in literature [1]. Such models represent biological regulations as a network where nodes represent elements of interactions, eg. genes, proteins, metabolites, while edges represent the presence of interaction activities between such network components. Four main network model architectures can be distinguished: i) information theory models, ii) boolean network models, iii) differential and difference equation models, iv) Bayesian models.

Information theory models correlate two genes by means of a correlation coefficient and a threshold. Two genes are predicted to interact if the correlation coefficient of their expression levels is above a threshold. For example, TD-ARACNE [2], ARACNE [3], and CLR [4] infer the network structure with a statistical score derived from the mutual information and a set of pruning heuristics.

Boolean networks use a binary variable to represent the state of a gene activity and a directed graph, where edges are represented by boolean functions, to represent the interactions between genes. REVEAL [5] is an algorithm that infers a boolean network model from gene expression data. Differential and difference equations describe gene expression changes as a function of the expression level of other genes. They are particular suitable to model the dynamic behavior of gene networks. The basic mathematical model of such approaches are a set of Ordinary Differential Equations (ODE) [6].

**Figure 1:**
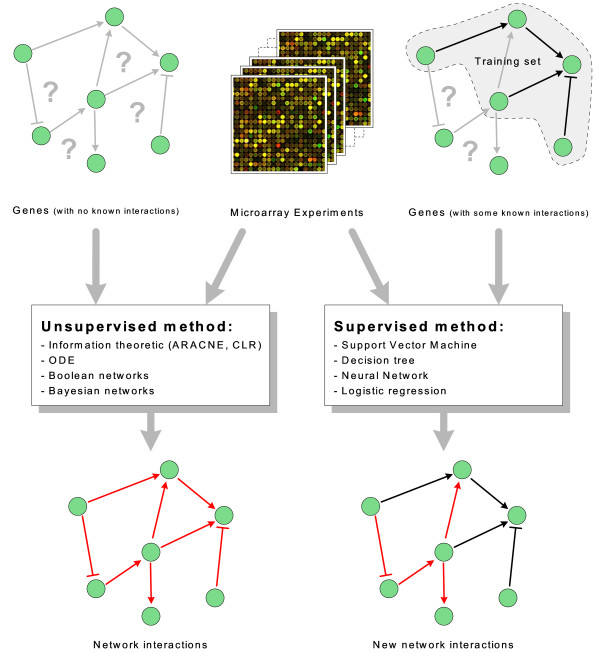
**Supervised vs unsupervised approaches in the identification of gene-gene interactions**. The figure depicts the two main perspectives followed by supervised and unsupervised methods in the inference of gene regulatory networks. Both induce the interaction model from genomic data (e.g. microarray experiments) but supervised methods need also a set of prior known interactions.

Bayesian models, or more generally graphical models, make use of Bayes rules and consider gene expressions as random variables. The major advantage is that the Bayesian framework allows for combining different types of data and prior knowledge in the process of gene networks inference [7]. For example, IRIS [8] is a software tool that infers the regulatory functions of a gene network by means of a factor graph model. Recently, supervised learning methods have been exploited to learn gene regulatory networks from gene expression data. They differ from the above mentioned unsupervised approaches in that they require as inputs not only gene expression data, but also a list of known regulation relationships, that act as a training set. Figure 1 depicts the main difference between supervised and unsupervised learning approaches. In machine-learning terminology, the method consists of building a binary classifier from the expression data of a set of prior known regulatory connections, available in public databases, and using such a classifier to predict new unknown connections. A selection of gene regulatory databases are: RegulonDB http://regulondb.ccg.unam.mx, TRRD http://wwwmgs.bionet.nsc.ru/mgs/gnw, KEGG http://www.genome.jp/kegg, Transfac http://www.gene-regulation.com, and IPA http://www.ingenuity.com. The necessity to know some regulations is not a serious restriction in many practical applications, as many regulations have already been characterized in model organisms (eg. *Escherichia coli*). The basic principle is to use the natural inductive reasoning to predict new regulations: if a gene A having expression profile *e*(*A*) is known to regulate a gene B with expression profile *e*(*B*), then all other couples of genes X and Y, having respectively expression profiles similar to *e*(*A*) and *e*(*B*) are likely to interact. Expression profiles play the role of feature vectors in the machine learning algorithm, while the output is a binary variable representing whether two genes interact or not. A similar idea has been proposed for the reconstruction of protein-protein interaction and metabolic networks. In [9] a combination of data sources has been used, including protein sequences, Gene Ontology annotations, local properties of the network, and homologous interactions in other species. In [10] the feature vector is built upon the sequence representation of proteins and metabolites. Instead, s feature vector composed of six different descriptors has been used in [11]: cysteine-cysteine coupling, 20 amino acid compositions, cysteine separation distance, cysteine ordering, protein molecular weight, and protein sequence length.

A large variety of machine learning algorithms have been proposed in literature and are available as working tools [12]. In the context of gene regulatory networks a first attempt has been made with Bayesian Networks, Linear Regression, Decision Trees, and Support Vector Machines (SVM) [13]. Among all the Support Vector Machine algorithm has attracted the attention of the bio-informatics community.

SIRENE [14] is the state-of-the-art method for the reconstruction of gene regulatory networks with a Support Vector Machine algorithm. The authors test SIRENE on a benchmark experiment of *Escherichia coli *genes composed by a compendium of gene expression data and a set of known regulations. A critical point of a binary supervised classifier algorithm is that the input consists normally of positive and negative examples. Actually, although prior known regulatory connections can safely be taken as a partial set of positive training examples, the choice of negative examples is not straightforward as no or few information is available regarding the fact that a given pair of genes are not interacting. The only available information is a partial set of interacting gene pairs, i.e. positive examples, and unlabeled data which could include both positive and negative examples. A common adopted solution is to consider all, or a random subset of, unlabeled examples as negative [14]. Whatever is the supervised algorithm, training with false negatives could affect the performance of the classifier, as it learns wrongly potentially positive examples as negatives. Learning from only positive and unlabeled data is a hot topic in the literature of data mining for the classification of web documents [15,16]. They differ from semi supervised learning, i.e. learning with a small set of labeled examples (both positive and negative), in the sense that the classification algorithm learns from a small subset of positive example and a huge set of unlabeled examples (both negative and positive). In literature two main classes of approaches can be distinguished:

• **Selection of reliable negatives**. The first class of approaches depends on a starting selection of reliable negative examples that usually depends on the application domain [17,18]. In [16] a two step strategy has been proposed in text classification domains: in the first step a set of reliable negative examples are selected from the unlabeled set by using the term frequency and inverse document frequency measure (td-idf); in the second step a sequence of classifiers are applied and then the best classifier is selected. In [19] a similar approach is used to predict non-coding RNA genes, where the first set of negative examples is built by maximizing the distances of negative sample points to the known positive sample points by using a distance metric built upon the RNA sequence. Such a negative set is iteratively refined by using a binary classifier based on current positive and negative examples until no further additional negative examples can be found. In [20] we proposed a method applied to gene regulatory networks that selects a reliable set of negatives by exploiting the known network topology.

• **Probability estimate correction**. The second class of approaches does not need labeled negative examples and basically tries to adjust the probability of being positive estimated by a traditional classifier trained with labeled and unlabeled examples. A general purpose method has been proposed in [15] where the authors show that, under certain circumstances, a classifier trained from only positive and unlabeled examples predicts probabilities that differ by only a constant factor from the true conditional probabilities of being positive. Such a result is used to show how to learn a classifiers from a non traditional training set.

In this paper we show that the probability estimation approach introduced in [15], called *PosOnly*, is a viable solution to the problem of learning gene regulatory networks without negative examples. It turns the problem of classify between positive and negative samples into the "simpler" problem of separating between labeled and unlabeled samples under the assumption that all the positive examples are randomly sampled from a uniform distribution. To this purpose we compare the *PosOnly *method with some recently proposed approaches to the supervised inference of regulatory networks: the traditional approach that considers unlabeled examples as negatives [14] (SVMOnly), and a method aimed at the selection of reliable negative examples [20] (PSEUDO-RANDOM).

## Methods

### PosOnly method

The *PosOnly *method has been introduced in [15] and works as follows. Let *x *be a feature vector and let *y *= {0, 1} and *s *= {0, 1} be binary labels. Let *s *= 1 if the example *x *is labeled, and let *s *= 0 if *x *is unlabeled. Positive examples are labeled, i.e. if *s *= 1 then *y *= 1, while unlabeled examples, *s *= 0, may be either positive *y *= 1 or negative *y *= 0. The goal of a probabilistic binary classifier is to learn *f*(*x*) such that *f*(*x*) = *p*(*y *= 1*|x*), i.e. the conditioned probability of being positive given a feature vector *x*. In [15] has been shown that *f*(*x*) = *p*(*s *= 1*|x*)/*p*(*s *= 1*|y *= 1) under the assumption that positive examples are labeled completely at random. The term *p*(*s *= 1*|x*) refers to a probabilistic binary classifier that learns from labeled and unlabeled data, while *p*(*s *= 1*|y *= 1) is an unknown constant which can be estimated empirically in various way. As stated in [15], this means that the conditional probabilities produced by a model trained on the labeled and unlabeled examples differ by only a constant factor from the conditional probabilities produced by a model trained on fully labeled positive and negative examples. Such result can be used to learn a probabilistic binary classifier, such as SVM (Support Vector Machine) with Platt scaling [21], using only positive and unlabeled data. The binary classifier is trained on labeled and unlabeled examples to get probability estimates *p*(*s *= 1*|x*). Such probabilities are then adjusted with the conditional probability *p*(*s *= 1*|y *= 1) computed empirically within a validation set *V *. Among the empirical estimations of *p*(*s *= 1*|y *= 1) proposed in [15], we used the following average:

where *V *is a validation set drawn in the same manner as the training set and *P *⊆ *V *is the set of labeled (i.e. positives) examples in *V *. A threshold, usually set to 0.5, discriminates if *x *belongs to the positive, *p*(*y *= 1*|x*) > 0.5, or negative, *p*(*y *= 0|*x*) > 0.5, class.

### PSEUDO-RANDOM method

**Figure 2:**
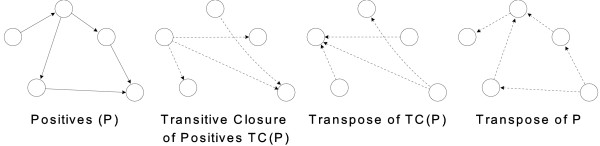
**Transitive closure heuristic example **[20]. The figure shows the PSEUDO-RANDOM negative selection heuristic based on the transitive closure of the known regulatory network.

The *PosOnly *method will be compared with a recently proposed method for selecting reliable negative examples proposed in [20] that works as follows. A gene interaction network can be modeled as a directed graph <*G, E *> where *G *represents the set of genes, i.e. nodes of the graph, and *E *represents the set of directed interactions between genes, i.e. edges of the graph. Let *P *⊆ *E *be the known gene-gene interactions, *Q *= *E - P *the unknown regulatory links, and *N *= *Complement*(*E*) the edges not contained in *E*. The unknown gene regulatory connections *Q *can be inferred by a machine learning scheme trained with the set of known regulatory connections. Precisely, *P *is the set of known positive examples, *N *is the set of all unknown negative examples and *Q *is the set of unknown positive examples. A selection of reliable negatives approach selects, from the unlabeled set *N *∪ *Q *of unknown connections, a sub set of reliable negative examples *S *which should be as much as possible composed of negative examples, i.e. *S *⋍ *N *and *S *∩ *Q *⋍⊘. Such negative examples are used to improve the training phase of a classifier. The *PSEUDO-RANDOM *method is built over the assumption that a regulatory network has no or few cycles and that it has a tree like structure. For complex eukaryote organisms such an assumption may not be true as many complex cell functions are based on homeostasis and feedback loops. In contrast, for simpler including *Escherichia coli *and *Saccharomyces cerevisiae*, such an assumption may be correct: there are unsupervised approaches, such as ARACNE, that prune the final network by removing 3-arc cycles [3]. This leads to an heuristic that selects as candidate negatives those given by the union of the transitive closure of the known network and its transpose. Figure 2 summarizes such an heuristic as:

where *TC*(*P*) is the transitive closure of *P*, i.e. the graph composed by the same nodes of *P *and the set of edges (*g*_*i*_, *g*_*j*_) such that there is a non-null path from *g*_*i *_to *g*_*j *_in *P *; while, *Transpose*(*X*) is the graph containing the edges of *X *reversed. Such a set is further extended with a small fraction of candidate negatives drawn randomly from *N *∪ *Q*.

### Research questions

In the following we detail: i) the research questions we aim at answering in this paper; and ii) the methods we followed to pursue such an aim. The main goal is to evaluate, by means of a benchmark experiment, the performances of the approaches, *PosOnly*, *PSEUDO-RANDOM *introduced in the previous Section, that address the problem of learning gene regulations with positive only data. Such approaches are then compared with a classifier trained with labeled and unlabeled examples (*SVMOnly*) and with the most widely used unsupervised information theoretic methods, ARACNE [3] and CLR [4]. In particular we aim at answering:

• **RQ1: ***How do PosOnly, PSEUDO-RANDOM, and SVMOnly performances vary with the percentage of known positives? *In particular, this research question aims to compare the performances of *PosOnly*, *PSEUDO-RANDOM*, and *SVMOnly *when the percentage of known positives varies from 10% to 100%.

• **RQ2: ***How do PosOnly, PSEUDO-RANDOM, and SVMOnly performances vary with the number of genes composing a regulatory network? *In particular, this research question aims to evaluate the performances of *PosOnly*, *PSEUDO-RANDOM*, and *SVMOnly *when network size varies from 10 to 500.

• **RQ3: ***How do PosOnly, PSEUDO-RANDOM, and SVMOnly performances compare with unsupervised information theoretic approaches, such as ARACNE and CLR? *In particular, this research question aims to compare supervised learning approaches, *PosOnly*, *PSEUDO-RANDOM*, and *SVMOnly*, with unsupervised information theoretic approaches at different network sizes and at different percentage of known positives.

The learning scheme, the datasets used, and the benchmark process to answer the above mentioned research questions are introduced in the following. To compare *PosOnly*, *PSEUDO-RANDOM*, *SVMOnly*, and unsupervised methods we performed a stratified 10-fold cross validation assuming different percentage of known positive examples within a gene regulatory network of size *G*. To perform an assessment a gold standard of the network is necessary. Simulated networks are widely used to test gene network inference algorithms as the complete set of gene-gene interactions is available. This is not true with experimental data where only a partial set of interactions is known from the literature and usually collected into public databases. This forces for different evaluation processes depending on which dataset, simulated or experimental, would be used.

### Learning scheme

For both *PosOnly *and *SVMOnly *we used the Support Vector Machine (SVM), with Platt scaling [21], to estimate the probability *p*(*s *= 1*|x*). In the case of *SVMOnly *such a probability is assumed to coincide with *p*(*y *= 1*|x*), instead, in the case of *PosOnly *such a probability is scaled with the empirical estimation *c *⋍ *p*(*s *= 1*|y *= 1) and then obtain *p*(*y *= 1*|x*) ⋍ *p*(*s *= 1*|x*) = *c*. For comparison purpose we used the Support Vector Machine with Platt scaling also for *PSEUDO-RANDOM *which is trained with the set of known positives and the set of negatives selected with the transitive closure heuristic.

We used the SVM implementation provided by LIBSVM, one of the most popular available tool [22]. The basic element of an SVM algorithm is a kernel function *K*(*x*_1_, *x*_2_), where *x*_1 _and *x*_2 _are feature vectors of two objects to be classified. In our case an object to be classified is a couple of genes, (A,B), represented with a feature vector composed by the concatenation of *e*(*A*) and *e*(*B*), i.e., (*e*(*A*)*; e*(*B*)) ∈ ℝ^2*n*^, the n-dimensional vectors of expression levels, standardized to zero mean and unit standard deviation, respectively of gene A and B. The idea is to construct an optimal hyperplane between two classes, +1 and -1, such that the distance of the hyperplane to the point closest to it is maximized. The kernel function implicitly map the original data into some high dimensional feature space, in which the optimal hyperplane can be found. A couple of genes, (A, B), classified as +1 means that gene A regulates gene B, instead, classified as -1 means that gene A does not regulate gene B. We used C-support vector classification (C-SVC) which solves the following problem:

subject to: *y*^*T*^*α *= 0

where *y*_*i *_∈ {+1, *-*1} is the class of vector *x*_*i*_; 0 ≤ *α*_*i *_≤ *C*; *i *= 1, ..., 2*n*; **e **is a vector with all elements equal to one; and *K*(*x*_*i*_, *y*_*j*_) is a kernel function. We adopted a radial basis kernel function defined as:

where *C *and *γ *are parameters that can be set empirically with a grid search cross validation [23].

### Benchmark process with simulated data

The process consists of the following three steps:

#### 1) Random generation of a gene-gene regulatory network of G genes

We generated simulated data with *GeneNetWeaver *http://gnw.sourceforge.net, a tool used to generate in silico benchmarks in the DREAM3 challenge initiative [24,25]. The *GeneNetWeaver *tool is able to obtain network topologies of a given size *G *by extracting randomly sub-networks from the gene-to-gene interaction networks of *Escherichia coli *or *Saccharomyces cerevisiae *(Yeast). The tool generates steady state levels for the wild-type and the null-mutant knock-down strains for each gene. This means that for a network of *G *genes there are *G *+ 1 experiments (wild-type and knock-down of every gene) leading to a feature vector composed of 2 × (*G *+ 1) attributes. The data corresponds to noisy measurements mRNA levels which have been normalized such that the maximum value in a given dataset is one. Auto-regulatory interactions were removed, i.e. no self-interactions are considered in the networks. As reported in the DREAM3 documentation, the tool takes great care to generate both network structure and dynamics that are biologically plausible.

**Figure 3:**
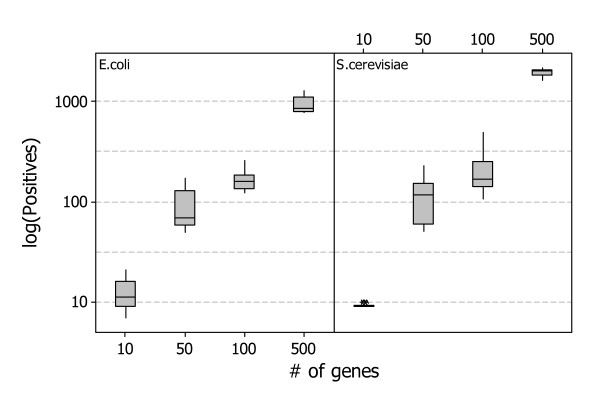
**Distribution of positives in the simulated datasets**. The figure shows the distribution of the number of positives in the set of random gene networks generated with the *GeneNetWeaver *tool.

**Figure 4:**
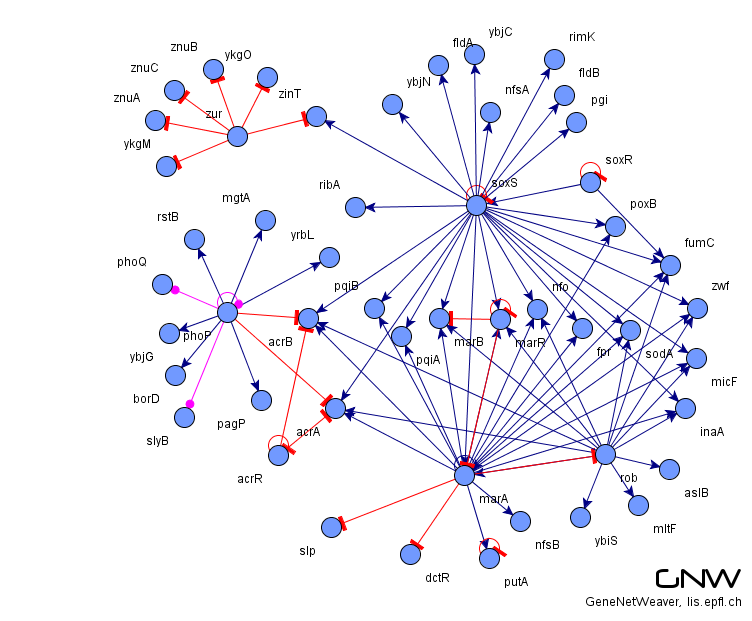
**An example of E. coli 50 gene sub-network generated with the GeneNetWeaver tool**. The figure shows a typical gene-gene interaction network generated with the *GeneNetWeaver *tool. The network is extracted randomly from the complete network of *E. coli *published in RegulonDB.

We generated for both *Escherichia coli *and *Saccharomyces cerevisiae *ten random gene interactions networks composed by *G *= 10, *G *= 50, *G *= 100, and *G *= 500 genes. Figure 3 shows the distribution of positives of the generated datasets we used in the benchmark process, while Figure 4 shows a typical gene regulatory network of 50 genes generated with the *GeneNetWeaver *tool.

#### 2) Random selection of P non-self interactions which are assumed to be known

This leads to a remaining set *Q *of non-self interaction assumed to be unknown, and *N *of all non-interactions. The fraction of with respect to is assumed to vary as: . In a learning scheme, *P *is the set of labeled, and positive, examples, and *Q *∪ *N *is the set of unlabeled examples. For each network of size *G*, the second step is repeated among ten random selection of *P *positives.

#### 3) Cross validation of PosOnly, PSEUDO-RANDOM, and SVMOnly classification performances

The validation consists of a stratified ten-fold cross validation and proceeds as follow. Partition P, Q, and N randomly into ten subsets each of roughly the same size (*P*_1_, *Q*_1_, *N*_1_), ..., (*P*_10_, *Q*_10_, *N*_10_). For each i-th partition a trial is performed with one subset reserved for testing (*P*_*i*_, *Q*_*i*_, *N*_*i*_), while the other nine subsets S for training the classifier. The training set is composed by the set of known labeled data, *P*_*i *_= ∪_*k*≠*i *_*P*_*k*_, S and the set *Q*_*i*_∪ *N*_*i *_= ∪_*k*≠*i *_*Q*_*k *_∪ *N*_*k*_, which simulate the unlabeled data. The i-th trial yields a confusion matrix as shown in Table 1, where *TP*_*i *_and *TN*_*i *_are, respectively, the number of positives and negatives correctly predicted by the classifier in the i-th trial; whereas *FP*_*i *_and *FN*_*i *_are, the number of false positives and false negatives in the i-th trial. The Precision (*PR*_*i*_) of positives, i.e. Positive Predictive Value, and the recall (*RC*_*i*_), i.e. Sensitivity, of the i-th trial are computed as:

**Table 1:** Confusion matrix of the i-th cross validation trial

	predicted	
actual	positive	negative
|*P*_*i*_∪ *Q*_*i*_|	*TP*_*i*_	*FN*_*i*_
|*N*_*i*_|	*FP*_*i*_	*TN*_*i*_

When no gene interactions are predicted (i.e. *TP*_*i *_+ *FP*_*i *_= 0) recall is zero and precision is assumed to be 1. The average indexes are computed among the ten trials as:  and . The tradeoff between precision and recall measures the effectiveness of a classifier. Among all we used the weighted harmonic mean of precision and recall, i.e. F-measure, as a measure that combines Precision and Recall as:

### Benchmark process with experimental data

To overcome computational limitation with the huge amount of experimental data we set up a benchmark process similar to the one adopted to evaluate the SIRENE supervised approach [14]. SIRENE predicts regulations in *Escherichia coli *by splitting the problem of regulatory network inference into many local binary classification subproblems, each associated with a Transcription Factor (TF). For each TF, we train an SVM classifier with a gaussian kernel to discriminate between genes known to be regulated and genes known not to be regulated by the TF, based on the expression patterns of such genes. The SIRENE inspired benchmark process we adopted with experimental data consists in the following steps:

#### 1) Selection of an experimental gene-gene network

As experimental data we used the expression and regulation data made publicly available by [26] of *Escherichia coli*, widely used in literature as an experimental benchmark [14]. The expression data, collected under different experimental conditions, consist of 445 *E. coli *Affymetrix Antisense2 microarray expression profiles for 4345 genes. Such data were standardized to zero mean and unit standard deviation. The regulation data consist of 3293 experimentally confirmed regulations between 154 TF and 1211 genes, extracted from the RegulonDB (version 5) database [27].

#### 2) Random selection of P* genes regulated by a given TF, assumed to be known

With experimental data, the complete set of gene-gene interactions is unknown and the partitions, *P*, *Q*, and *N*, cannot be simulated. Then, to differ them from the actual partitions referred above, we name such partitions as P*, the set of genes regulated by a given TF assumed to be known; Q*, the set of interaction assumed to be unknown; and N* the set of all non-interactions. The fraction of P* with respect to Q* is assumed to vary as: . In a learning scheme, P* is the set of labeled, and positive, examples, and Q* ∪ N* is the set of unlabeled examples. The second step is repeated for each TF among ten random selection of *P *positives.

#### 3) Cross validation of PosOnly, PSEUDO-RANDOM, and SVMOnly classification performances

The validation consists of a stratified ten-fold cross validation and proceeds as follow. Partition P*, Q*, and N* randomly into three subsets each of roughly the same size (*P*_1_, *Q*_1_, *N*_1_), ..., (, , ). For each i-th partition a trial is performed with one subset reserved for testing (, , ), and the other two for training the classifier. A cross validations of a classifier performance leads to precision and recall indexes, PR* and RC*, which need to be correctly interpreted. As P* ⊆ *P*, Q* ⊆ *Q*, and *N *⊆ N*, it is easy to see that PR* ≤ *PR *and . Hence, the value of precision, PR*, constitute a lower bound estimation of the actual precision, while the value of recall, RC*, can be correctly characterized when , which is the percentage of actually known gene-gene interactions, can be estimated in advance.

However, in domains such as *Escherichia coli *and *Saccharomyces cerevisiae *this can be assumed very high ( ~ 1), which means that the fraction of unknown of gene-gene regulations is very low.

### Selection of C and *γ *parameters

For experimental data we chose the same SVM parameters used by SIRENE [14], *C *= 1000 and *γ *= 1/128. For simulated data we selected the best SVM *C *and *γ *parameters following the procedure suggested in [23]. We performed parameter selection for each network size by using an independent set of 5 random gene networks for each organism (*Escherichia coli *and *Saccharomyces cerevisiae*). Those networks were used only for parameter estimation. Accuracy was evaluated with a different dataset. For each method and for each network, we performed stratified 10 fold cross validation with a grid of exponential sequences of *C *and *γ *values, as suggested in [23]. Test and training sets were the same for each method, the *C *parameter varied between 2^-5 ^and 2^15^, and *γ *varied between 2^-13 ^and 2^3^. We chose the parameters that give the best average F-measure for each method. Within a network, all methods exhibit the best performance with approximately the same parameter values. The *γ *parameter is more sensitive to network size, because of the number of attributes in the feature vector. Table 2 shows the selected *C *and *γ *parameters for each class of networks.

**Table 2:** The selected *C *and *γ *SVM parameters

Network size	*C*	*γ*
10	500	0.05
50	500	0.01
100	500	0.005
500	500	0.001

## Results and Discussion

In this section we discuss the results answering *RQ1*, *RQ2*, and *RQ3 *obtained in the context of simulated and, whereas possible, experimental data. To allow for replicability, raw data are available at the following url: *https://www.scoda.unisannio.it/rawdata/bmc-bioinformatics1009.tgz*.

### RQ1: How do PosOnly, PSEUDO-RANDOM, and SVMOnly performances vary with the percentage of known positives?

#### Results on Simulated data

**Figure 5:**
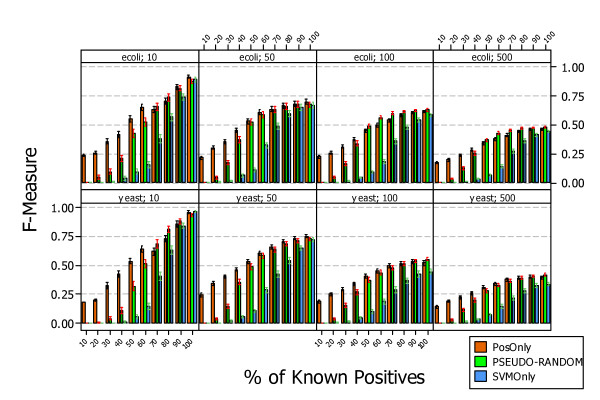
**Interval plots of F-Measure (95% CI of the mean) of PosOnly, PSEUDO-RANDOM, and SVMOnly classifiers on simulated Escherichia coli and Saccharomyces cerevisiae data**. The figure shows the interval plots with 95% confidence interval of *PosOnly*, *PSEUDO-RANDOM*, and *SVMOnly *F-Measure mean obtained on simulated data at different percentage of known positives. All algorithms exhibit a progressively increment in performance when the number of known positive examples grows from *P *= 10% to *P *= 100% reaching an almost convergent value at *P *= 100%.

Figure 5 shows the results, answering *RQ1*, obtained by applying the three approaches, *PosOnly*, *PSEUDO-RANDOM*, and *SVMOnly*, in the context of simulated data. All algorithms exhibit a progressively increment in performance when the number of known positive examples grows from *P *= 10% to *P *= 100% reaching an almost convergent value at *P *= 100%. This is due to the fact that when positive examples are all known all methods exhibit similar performances as the training set is composed by almost the same elements.

**Figure 6:**
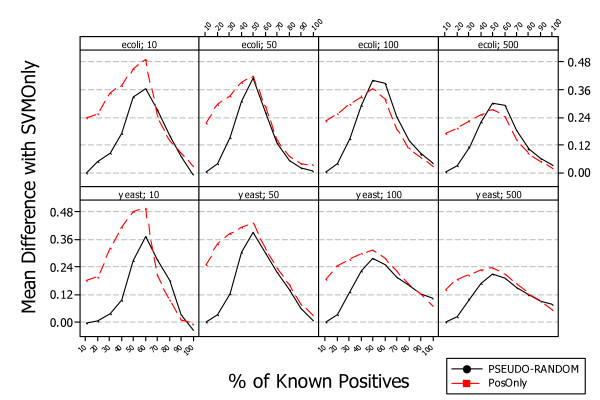
**Average difference between PosOnly, PSEUDO-RANDOM, and SVMOnly classifiers on simulated Escherichia coli and Saccharomyces cerevisiae data**. The figure shows the difference between *PosOnly*, *PSEUDO-RANDOM*, and *SVMOnly *F-Measure means at different percentage of known positives and with different network sizes. Such a difference varies with the number of genes and the maximum in the range around *P *= 40% and *P *= 60%.

Although the performance trend is the same in both organisms the absolute value could be different and it is lower for *S. cerevisiae *and higher for *E. coli*. This could be due to the fact that regulatory pathways of *S. cerevisiae *are more complex than those of *E. coli*. The positive gain obtained with *PosOnly *and *PSEUDO-RANDOM *is shown in Figure 6. The mean F-Measure difference with respect to *SVMOnly *is shown at different levels of known positives and for networks of different size. The mean difference varies with the number of genes and appears to be independent from the organisms. The maximum is reached at *P *= 60% for networks of size *G *= 10, at *P *= 50% for networks of size *G *= 50 and *G *= 100, and at 40% ≤ *P *≤ 50% for networks of size *G *= 500. A paired t-test shown that the difference is statistically significant in all simulated datasets, i.e. p-value < 0:01 for both methods *PosOnly *and *PSEUDO-RANDOM*. This is a promising results as it confirms the necessity to take into account that a partial knowledge of the gene regulatory network under investigation can lead to bad classifiers if it is not properly managed. Furthermore the difference is higher (p-value < 0.01) for *PosOnly *than *PSEUDO-RANDOM *especially for small networks and a low percentage of known positives.

#### Results on Experimental data

**Figure 7:**
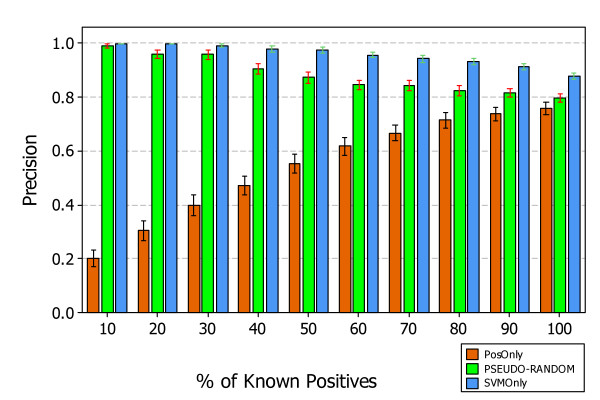
**Average Precision of PosOnly, PSEUDO-RANDOM, and SVMOnly classifiers on experimental data**. The figure shows the interval plots with 95% confidence interval of *PosOnly*, *PSEUDO-RANDOM*, and *SVMOnly *Precision mean values obtained on experimental data at different percentage of known positives.

**Figure 8:**
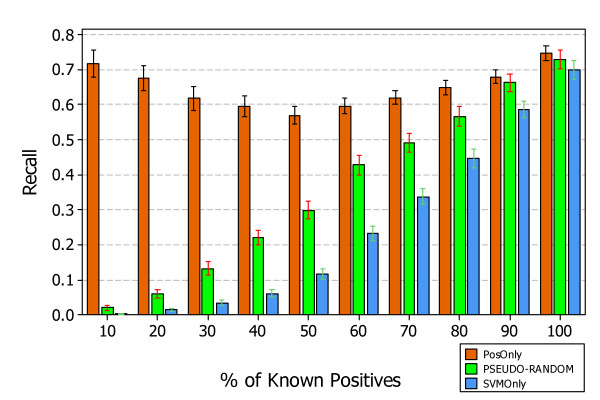
**Average Recall of PosOnly, PSEUDO-RANDOM, and SVMOnly classifiers on experimental data**. The figure shows the interval plots with 95% confidence interval of *PosOnly*, *PSEUDO-RANDOM*, and *SVMOnly *Recall mean values obtained on experimental data at different percentage of known positives.

**Figure 9:**
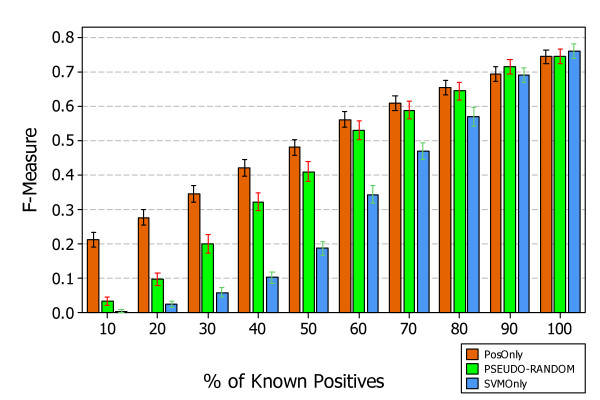
**Average F-Measure of PosOnly, PSEUDO-RANDOM, and SVMOnly classifiers on experimental data**. The figure shows the interval plots with 95% confidence interval of *PosOnly*, *PSEUDO-RANDOM*, and *SVMOnly *F-Measure mean obtained on experimental data at different percentage of known positives. Both algorithms exhibit a progressively increment in performance when the number of known positive examples grows from *P *= 10% to *P *= 100% reaching an almost convergent value at *P *= 100%.

Figures 7, 8, and 9 show the results, answering *RQ1*, obtained by applying the three approaches *PosOnly*, *PSEUDO-RANDOM*, and *SVMOnly*, in the context of experimental data.

Figures 7 and 8 show the precision and recall obtained at different percentage of known positives. The precision of *PSEUDO-RANDOM *and *SVMOnly *decrease with the percentage of known positives, instead their recall decrease showing a similar behavior, although *SVMOnly *exhibits a better precision while *PSEUDO-RANDOM *exhibits a better recall. The precision of *PosOnly *increases with the percentage of known positives but always lower than those exhibited by *PSEUDO-RANDOM *and *SVMOnly*. Instead, the recall of *PosOnly *is always higher than those exhibited by *PSEUDO-RANDOM *and *SVMOnly*. It decreases in the interval between *P *= 10% and *P *= 50%, reaching a minimum of 0:56, and then increases reaching a maximum of 0.76 at *P *= 100%.

Figure 9 shows the combination of precision and recall performance by means of F-Measure. It can be noticed that also in the experimental dataset all algorithms exhibit a progressively increment in performance when the number of known positives grows from 10% to 100% reaching an almost convergent value at *P *= 100%. *PosOnly *outperforms both *PSEUDO-RANDOM *and *SVMOnly *showing a statistically significant difference when the percentage of known positive is lower than *P *= 50%.

### RQ2: How do PosOnly, PSEUDO-RANDOM, and SVMOnly performances vary with the number of genes composing a regulatory network?

This research question can be answered only in the context of simulated data as in experimental data the number of genes cannot be varied.

#### Results on Simulated data

**Figure 10:**
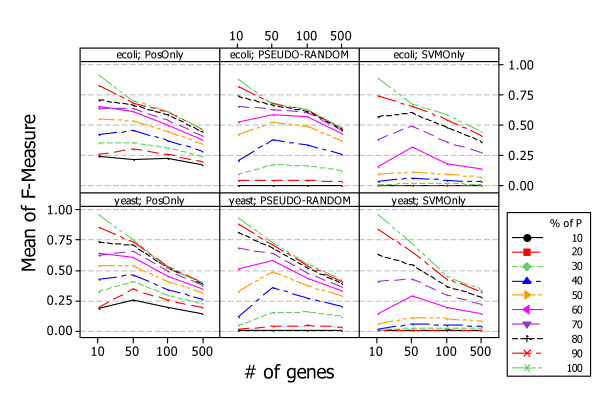
**Average F-Measure of PosOnly, PSEUDO-RANDOM, and SVMOnly on simulated data and at different network sizes**. The figure shows the performance of *PosOnly*, *PSEUDO-RANDOM*, and *SVMOnly *obtained with network of different sizes. Each approaches exhibit similar behavior when the number of genes increases: the average performance of the classifier increases when the percentages of known positives is low, while decreases when the percentages of known positives is high.

Figure 10 shows the results, answering *RQ2*, obtained by applying the three approaches, *PosOnly*, *PSEUDO-RANDOM*, and *SVMOnly*, in the context of simulated data. Both approaches exhibit similar behavior when the number of genes increases: the average performance of the classifier increases when the percentages of known positives is low, while decreases when the percentages of known positives is high.

### RQ3: How do PosOnly, PSEUDO-RANDOM, and SVMOnly performances compare with unsupervised information theoretic approaches, such as ARACNE and CLR?

#### Results on Simulated data

**Figure 11:**
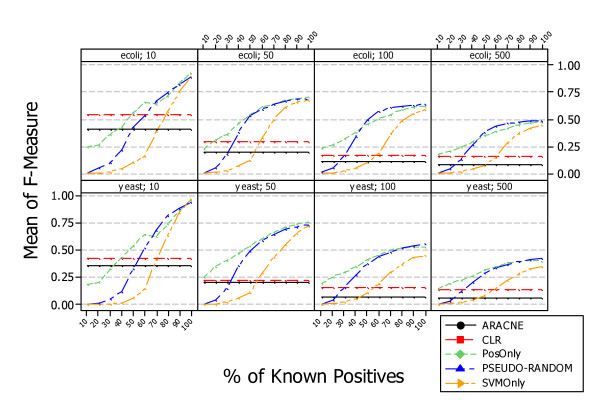
**Comparison with unsupervised methods, ARACNE and CLR in simulated data. Average F-Measure at different percentage of known positives**. The figure shows the difference between supervised and unsupervised methods obtained in the context of simulated data. The performance of supervised methods increases with the percentage of known positive examples. Instead, the performance of unsupervised information theoretic methods decreases with the number of genes in a regulatory network and is of course independent from the percentage of known positive examples. The intersection between supervised and unsupervised curves occur at different percentage of known positives and decreases with the number of genes composing the network.

Figure 11 shows the results, answering *RQ3*, obtained by applying *PosOnly*, *PSEUDO-RANDOM*, *SVMOnly*, and two unsupervised information theoretic methods, ARACNE and CLR, in the context of simulated data. Each figure shows the average F-measure at different percentage of known positives obtained with *PosOnly*, *PSEUDO-RANDOM*, *SVMOnly*, ARACNE, and CLR. As shown in previous research questions the performance of supervised learning methods increases with the percentage of known positive examples. Instead, the performance of unsupervised information theoretic methods decreases with the number of genes in a regulatory network and is of course independent from the percentage of known positive examples.

Figure 11 is particulary suitable to show the minimum percentage of known positives where the performance of learning methods starts to outperform the performance of unsupervised information theoretic methods, i.e. intersection between supervised and unsupervised curves. In can be noticed that *PosOnly *outperforms, or at least exhibit similar performances, at every percentage of known positives especially for large networks; while the intersection of *PSEUDO-RANDOM *and *SVMOnly *with unsupervised information theoretic methods curves occurs at different percentage of known positives. In particular such an intersections is dependent of the network size in both organisms and is lower for larger networks. This is mainly due to the fact that unsupervised methods works better with small networks making supervised methods more suitable form large networks.

**Figure 12:**
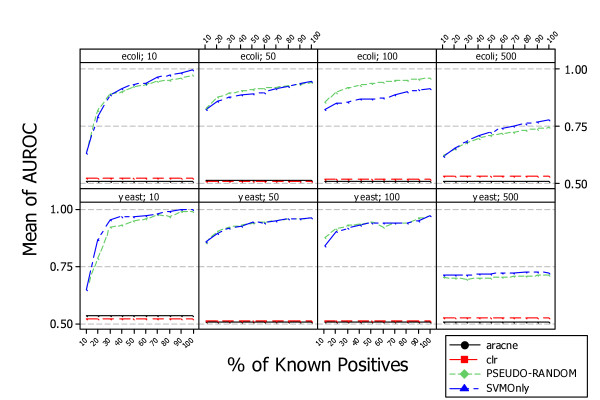
**Comparison with unsupervised methods, ARACNE and CLR in simulated data. Average AUROC at different percentage of known positives**. The figure shows the difference between supervised and unsupervised methods performance in terms of AUROC (Area Under the ROC curve) obtained in the context of simulated data. The AUROC of both *PosOnly *and *SVMOnly *is the same as the order of data predicted by each method is the same. It can be noticed that, similarly for F-Measure, the performance in term of AUROC of supervised methods increases with the percentage of known positive examples. Instead, the performance of unsupervised information theoretic methods are almost the same explaining the fact that unsupervised methods are able to select very precise top regulations but are unable to uncover (by means of a threshold) the complete set of gene regulations of a network.

Figure 12 shows the average AUROC (area under the ROC curve) measure obtained by scoring the predictions obtained with each approach. The AUROC measure does not depend on the cutoff threshold, but the F-Measure does. Therefore, the performance of *PosOnly *and *SVMOnly *is the same in terms of AUROC. The choice of a threshold is crucial to make a decision, and F-measure is a performance measure that takes into account this aspect of a classifier. Supervised methods outperform unsupervised ones when the percentage of known positives is low. This is because of the choice of threshold value, which in the case of supervised methods is incorrect when the number of known positives is low. In such a situation a supervised classifier makes incorrect classifications even though the scored list of predicted regulations has many true positives in the topmost positions. In contrast, a different threshold could not improve the performance of unsupervised methods, as shown by their AUROC measures

#### Results on Experimental data

**Figure 13:**
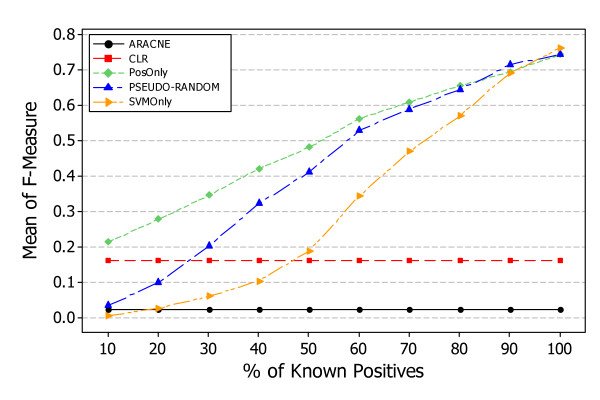
**Comparison with unsupervised methods, ARACNE and CLR in experimental data. Average F-Measure at different percentage of known positives**. The figure shows the difference between supervised and unsupervised methods obtained in the context of experimental data. The performance of supervised methods increases with the percentage of known positive examples. Instead, the performance of unsupervised methods is independent from the percentage of known positive examples.

Figure 13 shows the difference between supervised and unsupervised methods obtained in the context of experimental data. The performance of supervised methods increases with the percentage of known positive examples. Instead, the performance of unsupervised methods is independent from the percentage of known positive examples. *PosOnly *intersects the CLR curve at *P *= 50%, *PSEUDO-RANDOM *intersects the CLR curve at *P *= 40%, and *SVMOnly *intersects the CLR curve at *P *= 70%.

## Conclusions

We performed an experimental evaluation of a supervised learning algorithm, namely *PosOnly*, which is able to learn from only positive and unlabeled examples. Such a method is particulary suitable in the context of gene regulatory networks where a partial set of known regulatory connections is available in public databases. In such a contexts it is crucial to take into account that the only available information are a partial set of gene-gene interactions, i.e. positive examples, and unlabeled data which could include both positive and negative examples.

The data mining community developed a number of approaches to deal with such a problem. In this paper we adopted the approach introduced in [15] that we compared, through a benchmark experiment performed with simulated and experimental data, with a negative selection method introduced in [20] (*PSEUDO-RANDOM*) and with the current state of the art of supervised methods, namely *SVMOnly *[14]. We showed that *PosOnly*, outperforms significantly both methods *PSEUDO-RANDOM *and *SVMOnly *in simulated data, instead exhibit a slightly lower performance in experimental data. A comparison with unsupervised information theoretic methods has been performed showing that the performance of unsupervised information theoretic methods decreases drastically with the number of genes composing a regulatory network, instead the performances of *PosOnly*, *PSEUDO-RANDOM*, and *SVMOnly *decrease more slowly.

If one uses the *PosOnly *and *SVMOnly *methods to rank candidates, then the rankings should be the same. They are indeed the same in our experiments. In this case, the contribution of [15] is to show that the simple *SVMOnly *method actually is correct, something that is not obvious. At first sight the *SVMOnly *method is too naive as a solution to the positive-only problem; surprisingly, it is valid if all that is needed is a ranking of test examples.

If one wants to estimate probabilities for test examples, or if one wants to categorize candidates correctly at any given threshold (either 0.5 or some other value), then it is not correct to use probabilities produced by a standard classifier, whereas it is correct to use adjusted probabilities obtained with the *PosOnly *method. This happens, for example, if one wants to infer the overall gene regulatory network and a decision must be performed to classify the presence or absence of an arc between a pair of nodes/genes.

Note that the *PosOnly *method used in this paper is not the only valid way of obtaining correct probabilities. The paper [15] provides two other methods that are somewhat more complicated. In this research we use only the simplest method since it works well and will be easy for other researchers to apply. Any evaluation measure that is sensitive only to rank will indicate that the *PosOnly *and *SVMOnly *methods have equal performance. An example of such a measure is AUC, the area under the receiver operating characteristic (ROC) curve. However, measures that are sensitive to the correctness of conditional probabilities, for example mean squared error, will show that *PosOnly *performs better. Measures that are sensitive to the correctness of thresholds for making decisions, including F-Measure as used in our research, will also show that *PosOnly *performs better.

Results presented in this paper are partial and no general conclusions can be drawn. Threats to validity that can affect the results reported in the previous Section. In particular, our results can be affected by the limitations of the synthetic network generation tool and on the measurement errors in the experimental microarray data.

Threats to external validity, concerning the possibility to generalize our findings, affect the study although we evaluated the heuristics on two model organisms, and on a statistically significant sample of random regulatory networks. Nevertheless, analyses on further organisms are desirable, as well as the use of different simulated network generation tools. Instead, the study can be replicated as the tools are available for downloading, as well as simulated and experimental datasets. The benchmark process is detailed in Methods Section and we made raw data available for replication purposes.

Although more data is needed to validate empirically such results a biological validation is necessary to test the effectiveness of such approaches in real contexts. With respect to other gene network inference models, supervised methods need a set of known regulatory connection being available to learn the prediction model. As more genomic data become available such a limitation becomes less critical and we believe that machine learning methods could play a crucial role in the inference of new gene regulatory connections.

## Authors' contributions

LC conceived of the study, participated in its design and coordination, and drafted the manuscript. CE is the author of the PosOnly method applied in this paper and critically reviewed the manuscript. MC participated in the design and coordination of the study and contributed to draft the manuscript. All authors read and approved the final manuscript.
